# Medical educators’ beliefs about learning goals, teaching, and assessment in the context of curriculum changes: a qualitative study conducted at an Iranian medical school

**DOI:** 10.1186/s12909-021-02878-3

**Published:** 2021-08-24

**Authors:** Morteza Karami, Nooriyah Hashemi, Jeroen van Merrienboer

**Affiliations:** 1 Department of Curriculum Studies and Instruction, Faculty of Educational Sciences and Psychology, Ferdowsi University of Mashhad, Mashhad, Iran; 2 Faculty and Lecturer in English Department, Eshraaq University, Herat, Afghanistan; 3 School of Health Professions Education, Maastricht University, Maastricht, The Netherlands

**Keywords:** Medical educators’ beliefs, Learning goals, Teaching methods and assessment methods

## Abstract

**Background:**

Achieving changing needs, advancing knowledge, and innovations in higher education require the constant changes of medical school curricula and successfully applying the new reforms requires some modifications in the medical educators’ core beliefs. The purpose of this study was to describe the medical educators’ beliefs about the alignment of the learning goals with teaching and assessment processes in the context of the curriculum changes.

**Method:**

A qualitative method was used to study the medical educators’ beliefs through selecting the faculty participants via a purposeful sampling strategy. The study was conducted at a Medical School in Iran. For the individual interviews, we invited both the professors of the basic sciences and the clinical professors who had thought medical students for at least 5 years. Ten educators were interviewed.

**Result:**

The results of the research showed that, in the professors’ viewpoints, the development of competencies in the students has been abandoned and this is due to the priority of treatment to education in the clinical courses and the limited learning experiences. Moreover, the gap between the content and the context and the attendance of the students in the hospitals instead of the clinics to pass their internship courses has reduced the provision of authentic learning experiences. These conditions have affected the quality of education negatively. The non-systematic assessment has also worsened the situation.

**Conclusion:**

Despite the changes in the curriculum, the compartmentalization of the curriculum and the structure of the medical education have caused the professors’ beliefs to be in line with the past perspectives. Some modifications in the structure of the curriculum seem to be necessary.

## Background

Changes in education, including the curriculum and modes of teaching and learning, have been a major focus of higher education. Society’s changing needs, advancing knowledge [1], and innovations in education [2] require constant modifications of medical school curricula [3, 4]. In line with these changes, ongoing revision of the curricula based on problems and, more recently, competencies have become common. Earlier, curricula were often subject- or discipline-based [5], but, nowadays, they often focus on the problems or tasks that make an appeal on competencies that must be developed. This curriculum change has significant implications for teaching and assessment, which requires teachers to be engaged in more complex practices [6]. ‘Outcomes-based teaching and learning’ is a suitable approach to create alignment between the components of curriculum [7]. In this approach, competencies are the outcomes of learning, and learning experiences should be designed in a way that engages students in learning activities which lead to acquiring the intended outcomes. Assessment, then, examines how well those outcomes have been achieved by students [8]. Constructive alignment is a way curriculum developers and instructors can apply to create internal consistency between curriculum and instruction components. It is a form of outcomes-based teaching and learning in which both teaching and assessment are in line with the intended learning outcomes [7, 9].

In problem-based and competency-based curricula, learning goals are concentrated on the competencies that students must be able to apply in real world contexts, and teachers’ practices should, thus, require students to develop the competencies necessary to complete meaningful tasks and to solve problems [10]. Such complex tasks typically are better suited to a variety of competencies; that is, they require the integration of multiple objectives [11]. Whole-task approach to teaching [12, 13] provides an appropriate model for the development of the educational programs that help students learn and transfer professional competencies to an increasingly varied set of real-world contexts and settings. It also helps students in their future career endeavors [14]. Based on the theoretical models in this area such as the first principles of instruction model [15, 16] and the four-component instructional design model [17], the backbone of learning environment is to provide whole learning tasks based on real-world problems from simple to complex. In the process of performing these tasks, the learner is supported and this support is diminished and faded as the learner progresses. Learning and practicing in such environments provide a better context for learning transfer.

The competencies to be learned are introduced as early as possible through different learning tasks aligned with those competencies [18]. The final attainment levels are often described in terms of the standards that must be reached for each of the to-be-acquired competencies [13]. In outcomes-based teaching and learning, assessment is criterion-referenced. Students’ performances are assessed through judging them against established grading criteria or rubrics. In constructive alignment, the logic of assessment is holistic, not compartmentalized [7].

Implementation is critical to the success of a new curriculum. This step converts a mental exercise into reality [19]. Teachers, as the main agent in the educational change, play a vital role in the successful implementation of the new curricula [20]. Being responsible for enacting curriculum decisions, they hold beliefs that can support or undermine how learners experience these decisions and their activities are shaped by their beliefs [21]. Accordingly, teachers’ beliefs have been viewed as a key issue in the context of most educational reforms [22]. One possible explanation for the incomplete implementation of the ideal curriculum may be found in teachers’ beliefs [3]. Therefore, examining teachers’ beliefs is a vital area that needs to be addressed in the context of educational reforms [22].

Teachers, as all humans do, hold beliefs about a variety of topics, relationships, and processes [23] Their beliefs shape their real behavior towards their learners [24, 25]. Beliefs play a significant role in determining how educators organize knowledge and information, that is, they signify the most unchanging and least flexible dimension of an individual’s view on teaching’ [26]. Teachers’ beliefs refer to an integrated system of judgments that relate to teachers’ classroom work [27]. Their affect how teacher act in class and, thus, influence student learning [28].

In sum, teachers’ beliefs and practices play a key role in curricular reforms because teachers are the facilitators of educational change [29, 30], and policymakers, university leaders, department heads, and teacher educators must pay attention to teachers’ beliefs as part of any change efforts. Based on the mutual-adaptation, change is often (and should be) the result of adaptations and decisions made by users as they work with particular new policies or programs, with the policy or program and the user’s situation mutually determining the outcome [20]. The results of the research show, on one hand, teachers modify their beliefs and practices due to change processes; on the other hand, teachers changed the new curriculum in order to implement it more effectively [31].

Despite the growing trend towards recognizing the importance of teaching beliefs, little substantive research exists, specifically in medical education, about how medical educators interact with or implement the curricula. This lack of research is problematic for, at least, two reasons. First, it is common for medical faculty members to have little if any preparation for their role as teachers. Usually, their development as teachers is through a series of ‘trial by fire’ experiences via lectures, tutorials, and clinical teaching. They even spent little time reflecting on their underlying beliefs about teaching. Second, educators in a competency-based curriculum have different responsibilities. Their beliefs and practices should be aligned with learning goals; to reach those goals, educational interventions to teach complex tasks are needed [32].

Thus, the purpose of this study was to explore teachers’ beliefs regarding learning goals, teaching methods, assessment methods, and their interrelationships, as well as how these beliefs give meaning to their practices in the context of curriculum change. The specific questions guiding the study include: Do the teachers believe that the current teaching methods help the students acquire the desired competencies? Do the teachers believe that the current assessment methods are suitable to assess the competencies the students are expected to acquire? Do the teaching and assessment methods match each other and the realization of the desired competencies?

## Methods

This qualitative study was undertaken, using in-depth semi-structured interviews conducted on 10 medical educators of the general medicine program at the medical school of Mashhad University of Medical Sciences, Mashad, Iran. We used a phenomenology research method for this study. To identify and select the participants, a purposeful sampling strategy was used, as shown in Table 1. For the individual interviews, two groups of professors including the professors of basic sciences (such as biochemistry, physiology, and anatomy) and the clinical professors (the professors of externship and internship), who had instructed the medical students for at least 5 years, were invited.

**Table 1:** Characteristics of the participants

Educators (*N* = 10)	
Characteristic	Description
men	8
women	2
Professors of basic department	5
Professors of clinical department	5
Work Experience	More than 5 years
Age	40–60

### Study context

This study was conducted at a Medical School in Iran. In 2015, Iran’s Ministry of Health and Medical Education (MHME) announced that the institutions of higher education must ensure that all graduates of the medical programs can demonstrate professional commitment, decision-making, and problem solving (clinical skills), as well as communication skills, sensitivity to caring for patients, self-regulated skills for individual development or continuous learning, and the ability to improve community health. Recently, with an emphasis on expanding the role of family doctors, the re-design of the programs to prepare medical doctors has become more critical in the medical education system in Iran. To meet the new educational aims, a new curriculum was developed in 2017 by introducing the core competencies. Mashhad University of Medical Sciences is one of the large and advanced universities in Iran which is organized into 25 clinical and 19 basic departments. The research was conducted on the medical school curriculum revised in 2017–2018.

### Data collection

Given the goals of the current research and the revised curriculum characteristics, the interview statements were developed. Then, we conducted a pilot study on three participants to examine the content validity. The professors, who expressed their willingness to participate, were briefed about the interviews and the researchers’ reasons and interests for this study. They were then asked to attend the individual interviews after signing the informed consent forms. All interviews were conducted by the first author who is a professional expert at the curriculum studies. Moreover, no relationship was established between the interviewer and the participants prior to the study.

Data collection was conducted in two departments and 10 professors including five professors from the basic sciences department and five from the clinical department. The professors of the basic sciences were interviewed in the department of medical education at Mashhad University of Medical Sciences and the clinical professors were interviewed in Imam Reza Hospital. All interviews were conducted using the face-to-face method and no one else was present besides the participants and researcher during the interviews. All invited professors participated (participation rate of 100%) and none of the interviews were repeated. Each interview approximately lasted for 45 to 60 min. Notes were taken by the researcher and all interviews were recorded. After each individual interview, the content was checked to find out if new information had emerged. The interviews were then transcribed word by word. The transcripts were returned to the participants for the comments and corrections and the interviewing process continued up to the point of saturation. All data were saved confidential and were only accessible to the investigators.

### Data analysis

Given the Miles and Huberman’s theory [33], three successive phases of qualitative data analysis were applied to analyze the interviews. These phases include data reduction by coding, data structuring by categorization, and data interpretation by discussion, respectively [34]. All interview transcripts were transferred into the MAXQDA software package and all items were coded by NH. The codes were utilized as the first coding dictionary. MK revised the coding dictionary by removing the code duplicates and discussing the codes. MK and JvM organized the codes and discussed their organization to identify the different aspects of the learning goals, teaching, and assessment. During the analysis process, sub-themes were created and/or reduced by merging them. This allowed the analysis to reach internal homogeneity and external heterogeneity. The questioning and challenging of the emerging themes continued in an iterative process via the thematic analytical model by going back and forth between the researchers’ assumptions, ideas, questions and explanations and, then, a validation of these themes through comparing them with the interview texts. The analysis was continuously discussed and re-evaluated by the authors (MK, NH and JvM) to improve the reliability of the analysis through the examination of different dimensions, the contradictory information, and the interpretations. The participants were not asked to provide feedback on the findings. The data interpretation through discussion was the linking activity throughout the whole analysis process and during the decision-making process about the pertinent quotes.

## Results

In this section, we will describe the teachers’ beliefs about the alignment of the learning goals with the teaching and assessment processes. The interview data provided a detailed and clear understanding of the participants’ beliefs about the teaching in the medical contexts, and the results are discussed below:

### Limited active learning opportunities

The revised curriculum emphasizes the use of a variety of teaching methods by professors, including problem-based learning, task-based learning, outpatient-based education, community-oriented education, and so on. Nevertheless, most of these medical educators (7/10) believed learning opportunities were limited; even though, they were trying to point out the relevant and important information in the context to facilitate real learning. For example, Participant B2 stated:

*“The syllabuses in the course are defined and I use slides to present the lessons. I try to give clinical examples. For example, when talking about the enzymes, I talk about the anti-enzyme drugs that are related to the treatment of diseases. Because the students were doing their homework based on online copying and it had become something useless, I stopped giving homework to the students.”*

Also, Participant C3 recognized that:

*“The number of students is large, almost 100 people in the basic courses and there is no possibility of active methods such as discussion because there is no necessary interaction. The contents are mostly presented as lectures. Yet, this is a good form of presentation. If the teacher just read the slides, it would be worse.”*

According to most professors’ views, lack of time and the large volume of the materials lead them to employ the teaching methods that are more efficient and allow them to present more materials in the class.

### The ignorance of the Core competencies in the learning processes and assessment

The most important change in the new curriculum is the addition of professional competencies, but the professors (9/10), despite acknowledging the importance of this issue, believe that this has not been taken into account in the teaching-learning processes for various reasons, including the large number of students and the large volume of the materials.

Accordingly, Participant C1 stated:

*“The education system in the medical context is complex. A large number of students attend in one class and, in this situation, how a teacher can communicate well with all of them in a limited time and how can s/he manage them to acquire the core competencies?!”*

Accordingly, Participant B2 stated:

*“In different courses, we try to use the main up-to-date resources that are comprehensive and contain a lot of content, in teaching. It is very difficult to present all these materials during the semester sessions and, therefore, there is no opportunity for other learning activities.”*

Another reason is that the professors believe since there are specific courses for these competencies, it is not necessary to address them in other courses. For example, Participant B4 says:

*“We specifically have some courses in the curriculum to teach effective communication, professional ethics, and so on. Our students are supposed to acquire these skills there, and the mission of my course is something else. Therefore, we no longer need to address similar issues in different courses. For example, teaching thinking skills should also be included as a compulsory course in the curriculum; however, its teaching method can be different. One can talk about his experiences, one can show a film, but it should be one of the compulsory courses.”*

This is an example of compartmentalized thinking, because in a holistic approach it would make perfect sense to embed particular competencies in a variety of courses. What emerges from the interviewees’ opinions is that there is a large gap between the intended and implemented curriculum. Despite paying attention to these competencies in the intended curriculum document, the professors are not very faithful to it in practice (That is, in the implemented curriculum).

### The gaps between the content (class) and the context (workplace)

In the revised curriculum, the emphasis is on the integration of the basic and clinical courses in order to prepare students for professional responsibilities, but the medical educators (8/10) believed that the value of the contents is not well appreciated by the students because they have not been given the opportunities to put what they have learned into practice immediately. Participant B3 stated:

*“General physician students are not willing to take the basic sciences courses. When I myself was a medical student and I was going through an internship, I had the same belief as the current students. The belief is that the basic sciences are very inefficient courses that just has to be passed and finished to enter the physiopathology. What if the structure of a molecule has 4 rings?”*

In addition, Participant B5 explained:

*“Most of the students do not know about the philosophy of the subjects they are studying; however, some of the chosen contents are not much related to the real world that the students will experience in the context. The students study complex contents in their theoretical courses, but they do not have enough opportunities to acquire the needed competencies in their workplace.”*

Thus, while the content is primary, it needs to be put into practice in the context. These educators believed that one of their roles as university professors is to help the learners discern from the vast amounts of content knowledge the required information they need to apply to specific real-world cases in workplace. In addition, both the basic sciences educators and the clinical educators believed that the context of the medical school is more compartmentalized rather than holistic and there is a disconnection between the basic knowledge and the clinical sciences.

### Non-systematic assessments

In the new curriculum, the emphasis is on the use of various assessment methods such as written exams, oral exams, and computer interactive exams for theoretical courses, and portfolio, OSCE,^1^ OSLE,^2^ OSFE,^3^ and DOPS^4^ in clinical courses in order to assess students’ knowledge, skills, and attitudes more accurately. However, most professors (6/10) believed that due to the current conditions, the opportunity to use high-level tests is limited. The professors of the basic courses believed that the large number of students does not provide an option other than multiple-choice tests, and the professors of the clinical courses also consider their high workload as the main reason for this problem. The analyses of the data collected via the interviews highlighted the following issues:

*“The assessment is conducted based on the book content and we usually don’t give practical tests in the basic courses because applying practical tests in a crowded class is a complex process.”* (Participant B1)

*“The high workload in the ward and the large number of intern students in the ward prevent us from conducting a comprehensive and systematic assessment of the students.”* (Participant C2)

Most of the medical educators believed that the traditional method of assessment was used by most of the teachers. For example, Participant B2 remarked:

*“I feel that the knowledge of making tests is one of the ways that makes a teacher unique and the teachers should consider it. Unfortunately, we usually assess the students by giving them multiple-choice tests because the classes are crowded and it is difficult to check 100-150 papers at once; however, from the last year we have started giving open-ended tests to the students.”*

In spite of the importance of assessment in the educational system and the relative awareness of the professors of this issue, the organizational circumstances such as the large number of students, the high workload in the ward, have caused the professors to spend less energy on the accurate and systematic assessments of the students.

### Practicing in hospitals instead of clinics

The findings obtained from the medical educators (8/10) indicated that the GPs usually practice in the hospitals. This group of the medical educators found that practicing in the specialized hospitals is an important challenge for the GPs. They believed that the GPs needed to work in the clinics or specialized labs for their practical experiences. Participant C1 stated:

*“The educational system has been designed according to the MD system for both MD and GP students with the same contents. Although they study the same contents, their work positions are completely different.* After entering a specialized medical course, *MDs will have a good work position in future, with a high salary; however, the GPs don’t have such a good position.”*

Similarly, Participant C5 recognized:

*“On the practical level, students go to the specialized hospitals for internships, but these environments differ from the real work setting of a general practitioner. Working in a particular ward can assist the students to have a better understanding of that ward. It may also help them to plan their professional developments better. The educational system should consider the possible ways to prepare appropriate learning orientations for the GPs.”*

Most professors believed that the functional expectations of a GP are not learned in the various wards of a specialized hospital, and these learning environments pose a serious challenge in terms of authenticity.

### Clinical work preference to teaching

In the revised curriculum, in order to develop the specialized and core competencies, the new expectations have been defined for professors. The findings obtained from the educators’ beliefs (5/10) indicated that teaching competes with many other demands and takes place in a context that has constant distractions out of the control of the professors. Participant B4 explained:

*“I feel like I have so much stuff that I just want the students to understand, that I just want them to pass their course, and I don’t feel I have the time to teach efficiently. Some days, I should attend the clinics in the afternoons, so I am running back and forth.”*

Furthermore, Participant C5 stated:

*“Our hospital is the largest and most important hospital in the city and it is always full of patients who need care. For us, the patients are priorities, even in the educational rounds, because of the patients, we cannot pay enough attention to many aspects of education.”*

In each of these educators’ remarks quoted above, treatment is preferable to teaching. This has led to learning in clinical learning environments, which is not in line with the pre-designed goals and activities.

## Discussion

The current study focused on what the medical educators’ beliefs were about teaching, learning, and assessment. This research answered the following questions: Do the teachers believe that the current teaching methods help the students acquire the desired competencies? Do the teachers believe that the current assessment methods are suitable to assess the competencies the students are expected to acquire? Do the teaching and assessment methods match each other and the realization of the desired competencies?

The findings of the study are significant and show that the participants share a set of core teaching, learning, and assessment beliefs that shape their practices as teachers. Although the main change in the curriculum is the adoption of a competency-based approach, the results indicate professors believe that the development of competencies has not been considered in the implemented curriculum. They consider it as a result of the inadequate quantity and quality of experiences in the learning environment. The high volume of content and the large number of students in the basic courses and the priority of treatment over education in the clinical courses have caused the limited learning experiences to occur. However, the revised curriculum emphasizes the use of a variety of learning experiences, including problem-based learning, task-based learning, case studies, and the like. Given that there are ways to activate and involve students in large groups, it seems that the deficiencies in the specialty and competence of teachers in their teaching method have led to the formation of such beliefs, and this confirms the need to develop teaching skills for educators. Furthermore, the gap between the content and the context as well as the attendance in the hospitals instead of the clinics to pass their internship courses have decreased the provision of authentic learning experiences. These conditions have affected the quality of education negatively. The non-systematic assessment has also worsened the situation.

The medical educators believed that the gap between the theoretical contents and the real-world settings is one of the reasons to ignore the core competencies in the learning and assessment processes. This issue could be due to the fact that the basic courses such as physiopathology, anatomy, biochemistry, etc. and the clinical courses are presented in the form of H model.^5^ More specifically, in this model students pass all their basic courses inside the class without having any experiences in the practical courses and then go to the hospitals as interns. Following this model causes the gap between what the students learn in the class and what they need to know and, especially, do in the hospitals. Learning in such an educational climate is knowledge-based rather than competency-based.

It should also be pointed out that, in the first years, the focus is mainly on the basic sciences while in the subsequent years the clinical education and skill training are exclusively dealt with. In this way, learning is believed to be a simple accumulation of knowledge and the basic sciences are focused on in a preclinical phase, usually lasting 4 years. Every basic science is presented in an isolated course and there is little or no integration across disciplines [35].

The professors believed that in the clinical settings, treatment takes precedence over education. Of course, this situation seems to be due to the nature of the treatment environment. Irby et al. described the medical training as a long-term, inflexible and not learner-centered approach. Accordingly, the clinical learning settings have some specific characteristics that differentiate them from other learning settings. These characteristics include the combination of educational and work environment, high involvement with clinical affairs, and delegating tasks related to teaching to residents. These characteristics prevent professors from playing an effective teaching role. Paying attention to the commercial dimensions of health care has damaged the professional values of these educational environments. Moreover, the lack of a holistic approach causes a myopic view in students [36].

Despite their emphasis on content, the medical educators believed that the learning opportunities were limited, and assessment was also carried out in a non-systematic manner. These were the consequences of the lecture-dominated curriculum. In this approach, the learning processes were mainly the results of direct teaching. In a lecture-dominated curriculum with limited or no clinical experiences, students have few opportunities to observe the professional demeanor or actions of practitioners and, thus, have no role models to emulate. Later, as more laboratories and clinical experiences are introduced, there is still no formal focus on the development of the professional competencies and professional identity because this is probably one component of the curriculum versus the entirety.

The professors believed that the presence of students in the specialized hospitals made them unable to learn the skills required for a GP. To enhance the authenticity of the learning environments, students should attend clinics instead of hospitals. The use of authentic learning (i.e., connecting knowledge to real-world issues, problems, and applications) is a powerful learning strategy. Competency-based approach allows learners to practice the seven core skills, namely, clinical skills, communication skills, caring of patients, health development, individual development, professional commitment and decision making, and reasoning and problem solving [37]. If the development of these complex skills is integrated into the learning processes, learners will be more likely to transfer the skills to the real-world settings later. To this aim, task-based learning approach is a suitable choice. In task-centered learning environments, it is the real-world problems or tasks that drive learning [17, 38]. In creating such a learning environment, Kern’s six stages of curriculum development including 1) Problem identification and general needs assessment 2) Needs assessment for targeted learners 3) Goals and objectives 4) Educational strategies 5) Implementation 6) Evaluation and feedback can be of great help [19].

.In medical education, integration is important because the medical practice itself requires a great deal of integration. Integration refers to the connection of formally structured knowledge of the basic, clinical, and social sciences with clinical experiences in a much more balanced manner than it occurs today [39]. Integration promotes the blending of the basic sciences with each other, as well as with the clinical sciences. The benefits of integration are attributed to presenting information and problems in a way that mimics how they are encountered in the real world and presenting facts in relevant, meaningful, and connected ways. Integration should be viewed as a strategy of curricular design and development and; therefore, it should be considered at the program, course, and session levels [40].

## Conclusion

Curricular change is not merely concerned with the technical pedagogical dimension. Curriculum reform, particularly in an intricate system like a medical school, is full of problems such as resistance, power, inertia, and ego challenges, which necessitates a planned method, as well [41]. Teachers’ beliefs revealed the inconsistencies between the intended and implemented curriculum and the non-alignment of goals with the methods and assessment. The compartmentalization of the curriculum, the large number of students, the large volume of the instructional materials, and the nature of the clinical settings are the main causes of this incongruity. Therefore, to achieve an integrated learning context, the current teachers’ beliefs need to be further changed to realize more integrated learning in the future. They should connect the basic sciences to the clinical sciences and add the core competencies in their lesson plans. Moreover, the academic members of medical schools should revise the medical school curriculum by applying an inter-disciplinary approach, holding instructional seminars, and analyzing successful consequences to apply them as appropriate interventions.

### Strengths and limitations

We used a qualitative method to collect and collate the teachers’ beliefs in the learning goals, teaching methods, and assessment methods in the context of the curricular changes. To the best of our knowledge, this is the first research in medical education to address these issues.

Our study suffers from some important limitations. Firstly, as all participants were from a Family Medicine Department, their beliefs might not be generalized to the faculties in other fields and settings. Due to this limitation, further studies are recommended to be carried out to see whether similar perceived benefits and concerns will be discovered among teachers in different settings and fields of practice. Secondly, all teachers participating in this study were Iranian and their expression of feelings may differ from teachers in other cultures. It is suggested that the study be replicated in other cultures. Lastly, the potential for the interviewer’s bias might have influenced the views of the participants during the interviews. We attempted to reduce this possibility by limiting their dialogues to the questions and clarifications, as well as by instructing them to avoid expressing the opinions.

### Implications

Several implications arise from our study. Most of the medical educators believed that the learning outcomes and the assessment methods were not aligned with the curricular changes. Learning outcomes and assessment methods must be aligned; accordingly, integrated learning must be assessed in an integrated manner. Furthermore, a change towards more active methods and a whole-task approach to teaching and assessment are needed to help students acquire the desired skills. Finally, teachers should be given ongoing support, including the provision of feedback on their individual performances.

## Data Availability

Data will not be shared due to restrictions stipulated by the ethics committee when approving the study.

## Abbreviations

GP: General Practitioner
MD: Medical Doctor
MHME: Iran’s Ministry of Health and Medical Education

## Footnotes

1 Objective Structured Clinical Examination

2 Objective Structured Lab Examination

3 Objective Structured Field Examination

4 Directly Observed Procedural Skills

5 The traditional curriculum can be represented by an H shape structure, with all clinical sciences presented later in the curriculum. In this model, students study all their basic subjects inside the class and, then, continue the clinical sciences outside.
